# Expression of high-mobility-group-protein HMGI-C mRNA in the peripheral blood is an independent poor prognostic indicator for survival in metastatic breast cancer

**DOI:** 10.1038/sj.bjc.6600935

**Published:** 2003-04-29

**Authors:** C Langelotz, P Schmid, C Jakob, U Heider, K D Wernecke, K Possinger, O Sezer

**Affiliations:** 1 Department of Oncology and Hematology, University Hospital Charité, Humboldt-University, Schumannstr 20/21, 10098 Berlin, Germany; 2 Department of Medical Biometry, University Hospital Charité, Humboldt-University, Schumannstr 20/21, 10098 Berlin, Germany

**Keywords:** HMGI-C, metastatic breast cancer, independent prognostic factor

## Abstract

HMGI-C belongs to the high-mobility-group-protein (HMG) family of architectural transcription factors and considerable interest has recently been shown in its expression in neoplastic tissues and apparent involvement in tumorigenesis. We could previously demonstrate an expression of HMGI-C mRNA in the peripheral blood of breast cancer patients for the first time. In this prospective study, we evaluated the independent prognostic power of HMGI-C mRNA expression in the peripheral blood of an unselected cohort of 69 patients with metastatic breast cancer using a hemi-nested reverse transcriptase polymerase chain reaction (RT–PCR) followed by sequence analysis of the resulting PCR products. Multivariate analysis was performed using the Cox regression model. HMGI-C mRNA was detected in peripheral blood from 21 out of 69 (30%) patients with metastatic breast cancer. Median survival was 15.9 months in patients expressing HMGI-C, while in the group of patients without HMGI-C expression the median survival had not been reached yet after a median follow-up of 24.7 months and 85.4% were still alive in this group. Disease-specific survival was significantly worse for patients positive for HMGI-C in comparison to those not expressing HMGI-C (*P*=0.0001). In a multivariate regression analysis, HMGI-C remained an independent prognostic factor for overall survival (*P*=0.001) besides oestrogen receptor status (*P*=0.024) and presence of metastases in liver and lungs (*P*=0.029). HMGI-C expression in the peripheral blood of patients with metastatic breast cancer is a powerful independent indicator for poor overall survival and this is the first study to demonstrate its prognostic relevance in univariate and multivariate analysis.

Breast cancer is the most common malignancy affecting women. One area of intense breast cancer research has been in assessing prognostic factors of patient outcomes, and several molecular markers have been evaluated in association with established histologic and clinical prognostic parameters of breast cancer (Honkoop *et al*, 1998; Rizzieri *et al*, 1999; Sabbatini *et al*, 2000; Silva *et al*, 2001).

Considerable interest in HMGI proteins, a subfamily of the high-mobility-group-proteins, has been stimulated by observations that they are involved in the fundamental biological processes of cell proliferation and differentiationQ2 (Zhou and Chada, 1998). They serve as transcription factors and take part in the regulation of chromatin structure and function. HMGI proteins contain DNA binding domains and are involved in the assembly of the correct three-dimensional configuration of protein–DNA complexes (Tallini and Dal Cin, 1999). The HMGI family comprises three proteins: HMGI and HMGI(Y), which result from differential splicing from a single gene, and HMGI-C, which is encoded by a different gene, but shares some structural homologies with the former two (Bustin and Reeves, 1996; Goodwin, 1998; Chau *et al*, 1999; Tallini and Dal Cin, 1999). HMGI proteins facilitate gene activation through enhanceosome formation on inducible genes during embryonal development and within rapidly dividing cells. The family member HMGI-C was also shown to enhance the activity of the transcription factor NF-*κ*B (Mantovani *et al*, 1998). The HMGI-C gene is normally almost exclusively expressed during embryonic development and in haematopoietic stem cells in adults (Rogalla *et al*, 1996). Experiments with knockout mice having both HMGI-C alleles disrupted led to a pygmy phenotype in these mice (Zhou *et al*, 1995).

An expression of antisense HMGI-C RNA was shown to prevent retrovirally induced neoplastic transformation in rat thyroid cells by Berlingieri *et al* (1995). These findings and studies on HMGI-C expression in a variety of tumours (Chiappetta *et al*, 1995; Kazmierczak *et al*, 1996,^1998^; Staats *et al*, 1996; Rogalla *et al*, 1997,^1998^; Rommel *et al*, 1997; Giannini *et al*, 1999; Klotzbucher *et al*, 1999) have substantiated the important role of HMGI-C in the control of cell growth, differentiation and tumorigenesis. It could also be detected in the peripheral blood of patients with leukaemia, but no HMGI-C expression could be found in the peripheral blood samples of healthy donors (Rommel *et al*, 1997; Sezer *et al*, 2000). We could recently demonstrate for the first time that HMGI-C is expressed in the peripheral blood of breast cancer patients and that this expression is restricted to patients with metastatic disease (Sezer *et al*, 2000).

The purpose of the present study was to analyse the correlation between HMGI-C expression in the peripheral blood of metastatic breast cancer patients and clinicopathologic characteristics and this is the first study to evaluate the prognostic relevance of HMGI-C expression for survival using univariate and multivariate analysis.

## MATERIALS AND METHODS

Peripheral blood samples from an unselected cohort of 69 patients with metastatic breast cancer prior to the initiation of a chemotherapy were analysed in the present study. Institutional ethical approval was obtained and informed consent was given by the patients and healthy donors. Blood samples (5 ml) were immediately stabilised with DNA/RNA stabilisation reagent (Roche Diagnostics, formerly Boehringer Mannheim, Germany) after being drawn from the patient and processed according to the manufacturer's instructions.

### Reverse transcription–polymerase chain reaction for assessing the expression of HMGI-C mRNA

Briefly, mRNA was obtained using an mRNA kit (mRNA Isolation Kit, Roche Diagnostics, formerly Boehringer Mannheim, Germany), cDNA was synthesised using the adapter primer (AP2) and Superscript II reverse transcriptase (RT) (Life Technologies, Eggenstein, Germany) and HMGI-C expression was determined using a hemi-nested reverse transcription–polymerase chain reaction (RT–PCR), as previously described (Rogalla *et al*, 1996; Sezer *et al*, 2000) and confirmed by DNA sequencing (ABI Prism DNA sequencer, Perkin-Elmer).

### Statistical analysis

All statistical analyses were performed using SPSS software (Version 10.0, SPSS Inc, Chicago, IL, USA). Possible differences in clinicopathologic characteristics of the patient groups with and without circulating HMGI-C mRNA were analysed using exact *χ*^2^- and Mann–Whitney tests. The Kaplan–Meier method was used to estimate survival rates and log-rank tests were applied to compare survival curves between two groups. Cox multivariate regression analysis was used to assess the prognostic significances of the various factors for overall survival, with variables entering the model if changes in minus twice the log likelihood were statistically significant at the 5% significance level. Proportional hazard was tested with the log minus log plot.

### Results

Expression of HMGI-C mRNA in the peripheral blood was detected in 21 (30.4%) of the 69 patients with metastatic breast cancer. Patient characteristics according to HMGI-C expression status are listed in Table 1

**Table 1:** Association of HMGI-C expression with clinicopathologic characteristics of patients

	**HMGI-C expression**		
**Variables**	**Positive (%)**	**Negative (%)**	***P*-value^a^**
*Age* (*years*)			
Mean±s.d.	55.6±7.8	56.0±13.1	0.80
Range	39.9−71.9	32.1−85.1	
Median	55.3	58.4	
*Time from diagnosis to first metastatic event* (*months*)			
Mean±s.d.	27.1±19.8	31.0±31.0	0.87
Range	0.0−85.0	0.0−120.0	
Median	23.0	25.5	
*Menopausal status*			0.18
Premenopausal	0 (0)	5 (10.4)	
Postmenopausal	19 (90.5)	38 (79.2)	
Unknown	2 (9.5)	5 (10.4)	
*Histology*			1.00
Ductal carcinoma	20 (95.2)	45 (93.8)	
Lobular carcinoma	1 (4.8)	3 (6.2)	
*Oestrogen-receptor status*			0.78
Positive	11 (52.4)	27 (56.2)	
Negative	8 (38.1)	16 (33.3)	
Unknown	2 (9.5)	5 (10.4)	
*Progesterone-receptor status*			0.79
Positive	8 (38.1)	19 (39.6)	
Negative	12 (57.1)	24 (50.0)	
Unknown	1 (4.8)	5 (10.6)	
*Site of metastases*			
Any visceral	19 (90.5)	39 (81.3)	0.48
Lung	12 (57.1)	25 (52.1)	0.80
Liver	12 (57.1)	20 (41.7)	0.29
Lung and Liver	5 (23.8)	8 (16.7)	0.52
Bone	14 (66.7)	21 (42.6)	0.12
Soft tissues	5 (23.8)	13 (27.1)	1.00
*Chemotherapy*			
Ever	16 (76.2)	29 (60.4)	0.28
Adjuvant	12 (57.1)	20 (41.7)	0.30
Previous palliative	7 (33.3)	10 (20.8)	0.36
Anthracyclines	9 (42.9)	19 (39.6)	1.00
Taxanes	7 (33.3)	10 (20.8)	0.37
*Endocrine therapy*	12 (57.1)	29 (60.4)	1.00
Tamoxifen	11 (52.4)	26 (54.2)	1.00
Letrozole	3 (14.3)	13 (27.1)	0.36
*Radiation therapy*	12 (57.1)	22 (45.8)	0.44

a *p*-values for age and time from diagnosis to the first metastatic event were obtained using the Mann–Whitney test; other *p*-values were derived using the *χ*^2^-test.

**Figure 1:**
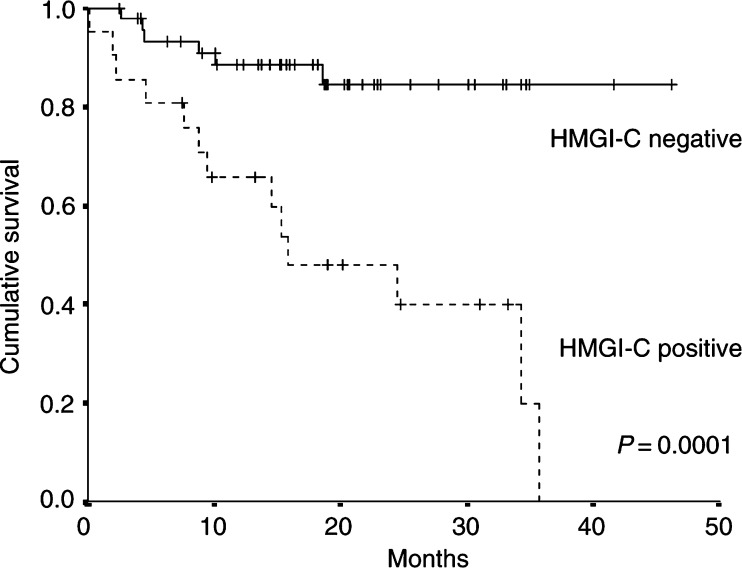
Kaplan–Meier curves for disease-specific survival of 69 metastatic breast cancer patients with detectable HMGI-C expression (HMGI-C positive, *N*=21) *vs* patients in whom HMGI-C could not be detected (HMGI-C negative, *N*=48) by RT–PCR in peripheral blood.

. There was no statistically significant difference between the group of patients positive for HMGI-C expression and the group of patients without detectable HMGI-C in terms of age, time from diagnosis to first metastatic event, menopausal status, histologic type, hormone receptor status and sites of metastases. The two groups did not differ with respect to having ever received chemotherapy, having received adjuvant or palliative chemotherapy, treatment with taxanes or anthracyclines, endocrine therapy or radiation therapy. Median length of follow-up was 24.7 months for all patients. Median survival was 15.9 months in patients expressing HMGI-C, 38% were still alive at the last follow-up contact, while in the group of patients without HMGI-C expression the median survival had not been reached yet, and 85.4% were still alive at last follow-up contact (*P*=0.0001). Kaplan–Meier analysis showed that patients with HMGI-C expression had statistically significant worse overall survival (*P*=0.0001) (Figure 1

). Cox multivariate regression analysis performed after univariate analyses identified HMGI-C to be an independent poor prognostic factor for patient survival (*P*=0.001). In addition, positive oestrogen receptor status was significant for better overall survival (*P*=0.024) and presence of metastases in both liver and lung was significant for worse outcome (*P*=0.029) (Table 2

**Table 2:** Prognostic significance of HMGI-C mRNA in peripheral blood and other variables associated with survival as determined by univariate analysis

**Variables**	***P*-value**
HMGI-C expression	0.0001
Oestrogen receptor status	0.055
Progesterone receptor status	0.49
Menopausal status	0.39
Liver and lung metastases	0.10
Sites of metastases >2	0.16
Adjuvant chemotherapy	0.20

and Table 3

**Table 3:** Multivariate analysis using the stepwise Cox regression model for overall survival, final model

	**Multivariate analysis**		
**Variables**	**Hazard ratio**	**95% CI**	***P***-value
HMGI-C expression	6.38	2.22–18.34	0.001
Estrogen receptor positivity	0.31	0.11–0.86	0.024
Liver and lung metastases	3.46	1.13–10.58	0.029

).

## DISCUSSION

HMGI-C is an architectural transcription factor and plays a key role in important cellular processes such as DNA transcription in rapidly dividing embryonal cells and in neoplastic cells (Tallini and Dal Cin, 1999). It was found to be expressed in a variety of tumour tissues, but not in normal tissue adjacent to the tumour (Rogalla *et al*, 1996). In tumour tissues, a correlation between HMGI-C expression and grading has been reported, and in a study on HMGI-C expression in breast cancer tissues, HMGI-C was predominantly noted in tumours with high histological grade (Rogalla *et al*, 1997). Berlingieri *et al* (1995) demonstrated the appearance of a highly malignant phenotype in relation with HMGI-C expression in differentiated rat thyroid cells transformed with oncogenes. V-mos and v-ras-Ki oncogenes were implied to induce the synthesis of HMGI protein, and the block of this induction by the presence of an HMGI-C antisense construct could be shown to inhibit cell transformation. HMGI-C did not behave like a classical transforming oncogene though, since when transfected in normal thyroid cells, it did not cause their transformation, thus suggesting that its expression is necessary but not sufficient to achieve the transformed phenotype (Berlingieri *et al*, 1995). Neoplastic transformation was associated with a drastic increase in AP-1 activity, which was blocked by the suppression of HMGI protein synthesis. The absence of AP-1 transcriptional activity induction, directly or indirectly regulated by the HMGI proteins, would inhibit the expression of AP-1-dependent genes such as VEGF, collagenase I and stromelysin, which are required for neoplastic cell transformation (Vallone *et al*, 1997). It was noted that the high intracellular levels of HMGI proteins in transformed malignant cells are not simply the result of increased cellular proliferation. In fact, nontransformed cells proliferating at approximately the same rate as their transformed counterpart expressed consistently lower HMGI-C or HMGI(Y) (Tallini and Dal Cin, 1999). A recent study by Scala *et al* (2001) showed though, that in pygmy mice carrying a disrupted HMGI-C gene, induction of thyroid carcinomas with radiation or the E7 papilloma virus oncogene took place with the same frequency as in wildtype mice, therefore indicating that HMGI-C is not necessarily required for *in vivo* thyroid carcinogenesis (Scala *et al*, 2001). It was considered that HMGI(Y) proteins, rather than HMGI-C, may be required for thyroid cell transformation.

Similar observations as for HMGI-C concerning its expression in association with grade and stage of tumours were made for HMGI(Y), given its 50% amino-acid sequence homology with HMGI-C (Tallini and Dal Cin, 1999). In a study on colorectal neoplastic tissues a correlation could be found between an increased HMGI(Y) protein expression because of an increase in its mRNA and various clinicopathological parameters, known to be indicative of a poor prognosis (Abe *et al*, 1999). A significant correlation between HMGI(Y) mRNA expression and tumour grade and stage could be found in another study on prostate cancer (Tamimi *et al*, 1996). The above data demonstrate that HMGI-C and HMGI(Y) are important elements in tumorigenesis, while a precise definition of their role in tumour initiation and progression is still missing. So far studies on the expression of HMGI-C had focused on tumour tissues and cell lines and only one study was available on HMGI-C expression in the peripheral blood of leukemia patients, until we could recently demonstrate that HMGI-C is expressed in the peripheral blood of a subset of patients with breast cancer. This expression was restricted to patients with metastatic disease.

In the present study, we evaluated the prognostic relevance of HMGI-C expression in the peripheral blood of breast cancer patients with metastatic disease with respect to clinicopathologic parameters. We could show for the first time that HMGI-C expression is highly significant for worse outcome in univariate analysis, and we could furthermore demonstrate in multivariate analysis that circulating HMGI-C mRNA is a powerful independent prognostic indicator for overall survival. The significance of HMGI-C mRNA detection in peripheral blood, even allowing for the relatively small number of patients with metastatic breast cancer studied here, implies that this is a particularly strong prognostic factor. The multivariate analysis also showed that the presence of lung and liver metastases was significantly associated with worse outcome, which is consistent with previous studies, as is the fact that patients who were oestrogen receptor positive had significantly better overall survival (Rizzieri *et al*, 1999).

Reverse transcription–polymerase chain reaction of peripheral blood for HMGI-C mRNA, identifying those patients with metastatic breast cancer with an unfavourable prognosis, could be of diagnostic importance complementary to current methods for assessment of disease status and prediction of outcome, reflecting distinct biologic features of the disease. So far no expression of HMGI-C in the peripheral blood of healthy controls could be found, which is of advantage, since the specificity of other RT–PCR assays in breast cancer, such as RT–PCR for cytokeratin 19, had to be questioned due to an expression in healthy controls (Bostick *et al*, 1998; Slade *et al*, 1999).

The additional information gained by RT–PCR for HMGI-C may help to improve treatment strategies in allowing for a selection of breast cancer patients with metastatic disease with a worse prognosis. Further studies are needed to evaluate tailored therapy options for this risk group of patients with a particularly poor prognosis.
